# Biocompatibility and antioxidant activity of a novel carrageenan based injectable hydrogel scaffold incorporated with *Cissus quadrangularis*: an in vitro study

**DOI:** 10.1186/s12903-022-02409-6

**Published:** 2022-09-05

**Authors:** Sruthi Sairaman, M. S. Nivedhitha, Deepti Shrivastava, Meshal Aber Al Onazi, Hmoud Ali Algarni, Mohammed Mustafa, Ali Robaian Alqahtani, Nouf AlQahtani, Kavalipurapu Venkata Teja, Krishnamachari Janani, Rajalakshmanan Eswaramoorthy, M. P. Sudhakar, Mohammad Khursheed Alam, Kumar Chandan Srivastava

**Affiliations:** 1 Department of Conservative Dentistry and Endodontics, Saveetha Dental College and Hospitals, Saveetha Institute of Medical and Technical Sciences, Chennai, Tamil Nadu India; 2 Periodontics, Preventive Dentistry, College of Dentistry, Jouf University, Sakaka, 72345 Saudi Arabia; 3 Department of Operative Dentistry and Endodontics, College of Dentistry, Jouf University, Sakaka, 72345 Saudi Arabia; 4 Department of Conservative Dental Sciences, College of Dentistry, Prince Sattam Bin Abdulaziz University, Al-Kharj, 11942, Saudi Arabia; 5 Dental Department, FM & PHC, NGHA-CR, Riyadh, Saudi Arabia; 6 Department of Conservative Dentistry and Endodontics, Mamata Institute of Dental Sciences, Bachupally, Hyderabad, Telangana state 500090 India; 7 Department of Conservative Dentistry and Endodontics, SRM Dental College, SRM Institute of Science and Technology, Chennai, India; 8 Department of Biomaterials, Saveetha Dental College and Hospitals, Saveetha Institute of Medical and Technical Sciences, Chennai, Tamil Nadu India; 9 Department of Orthodontics, Department of Preventive Dentistry, College of Dentistry, Jouf University, Sakaka, 72345 Saudi Arabia; 10 Oral Medicine and Maxillofacial Radiology, Department of Oral and Maxillofacial Surgery and Diagnostic Sciences, College of Dentistry, Jouf University, Sakaka, 72345 Saudi Arabia

**Keywords:** Scaffold, Hydrogel, Carrageenan, *Cissus quadrangularis*, Regeneration, Dentin–pulp complex, Antioxidant activity, Tissue engineering

## Abstract

**Background:**

Over the past years, polysaccharide-based scaffolds have emerged as the most promising material for tissue engineering. In the present study, carrageenan, an injectable scaffold has been used owing to its advantage and superior property. *Cissus quadrangularis*, a natural agent was incorporated into the carrageenan scaffold. Therefore, the present study aimed to assess the antioxidant activity and biocompatibility of this novel material.

**Methods:**

The present in vitro study comprised of four study groups each constituting a sample of 15 with a total sample size of sixty (n = 60). The carrageenan hydrogel devoid of *Cissus quadrangularis* acted as the control group (Group-I). Based on the concentration of aqueous extract of *Cissus quadrangularis* (10% w/v, 20% w/v and 30% w/v) in carrageenan hydrogel, respective study groups namely II, III and IV were considered. Antioxidant activity was assessed using a 1,1-diphenyl-2-picrylhydrazyl radical scavenging assay, whereas the biocompatibility test was performed using a brine shrimp lethality assay. The microstructure and surface morphology of the hydrogel samples containing different concentrations of *Cissus quadrangularis* aqueous extract was investigated using SEM. One-way ANOVA with the post hoc tukey test was performed using SPSS software v22.

**Results:**

A significant difference (*P* < 0.05) in the antioxidant activity was observed among the study groups. Group III reported the highest activity, whereas the control group showed the least antioxidant activity. Additionally, a significant (*P* < 0.01) drop in the antioxidant activity was observed in group IV when compared with group III. While assessing the biocompatibility, a significant (*P* < 0.001) dose-dependent increase in biocompatibility was observed with the increasing concentration of aqueous extract of *Cissus quadrangularis*. SEM analysis in group III showed even distribution throughout the hydrogel although the particles are close and densely arranged. Reduced antioxidant activity in group IV was probably due to clumping of the particles, thus reducing the active surface area.

**Conclusion:**

Keeping the limitations of in vitro study, it can be assumed that a carrageenan based injectable hydrogel scaffold incorporated with 20% w/v *Cissus quadrangularis* can provide a favourable micro-environment as it is biocompatible and possess better antioxidant property.

## Background

In recent days, there is an immense focus on recreating the lost architecture of human tissue or defective tissues; however, it’s quite challenging [1]. Procedures such as harvesting a graft for regenerative purposes could lead to serious complications including pain, morbidity and a higher risk of infection [1]. Hence, tissue engineering principles are employed for repair, regeneration and enhancing the function of defective tissues.

The success of tissue regeneration depends on the material of choice. In this context, natural scaffold such as hydrogel claims to be a valid treatment option [2] as they carry a lower risk for cytotoxicity [3, 4]. They have a unique three-dimensional polymeric network wherein water is the main liquid component. The hydrophilic nature of these hydrogels helps in retaining the higher fluid content and thus allowing the diffusion of nutrients through their structure [5]. They are biocompatible with adjustable mechanical properties and their cross-linking structure renders them less soluble despite high-water concentrations [6]. They have a gelatinous structure, which provides essential cell support, along with their ability to get loaded with various drugs, making them a good drug delivery system [5, 7, 8]. Despite being biodegradable, hydrogels also release the bioactive molecules influencing the surrounding environment [9, 10]. In addition to being a carrier for the cells or bioactive molecules, they are mainly applied as a space-filling material for tissue engineering [11]. Among various hydrogel formulations [12], polysaccharide-based hydrogels have shown promising results in tissue engineering [13]. Being thixotropic, they can be injected into the targeted space without altering their physical, mechanical or biological properties [13].

Carrageenan-based hydrogel formulation is one such naturally occurring sulphate polysaccharide-based formulation having versatile properties [14]. Carrageenan is a natural compound obtained from red seaweed, which is a marine red alga [15]. Structurally, carrageenan has a resemblance to the glycosaminoglycans which form the extracellular matrix (ECM) of tissue, hence in physiological conditions, injecting this scaffold into the tissue defect will offer added benefits [16]. Studies have found that carrageenan-based extract is useful in the controlled delivery of drugs [17], bone tissue engineering purposes [18] as well as in wound healing [19].

Promising results have been documented with plant-derived bioactive compounds and their secondary metabolites in regenerative and therapeutic tissue engineering applications [20]. *Cissus quadrangularis* is a vitaceae plant that has been used as a medicinal herb in India and Africa for ages. In traditional medicine, it is used for its antibacterial, analgesic, anti-inflammatory and antioxidant properties. Along with this, it is employed for bone fracture healing and the prevention of osteoporosis [21]. Additionally, Calcium (about 4% by weight) and phosphorus ions are abundant in the *Cissus quadrangularis* stem extract [22].

In tissue engineering, as we are focused on achieving a favourable environment that could induce the claimed odontogenesis and osteogenesis, currently studied extract, being anti-inflammatory along with being antioxidant, could favour the environment for biomineralization. For a material to be used in tissue engineering, it needs to be biocompatible and antioxidant to prevent oxidative stress thereby inhibiting the damage caused by free radicals. Thus, the current study aimed at evaluating the activity of the *Cissus quadrangularis* incorporated in carrageenan-based injectable hydrogel as a natural scaffold. The present study is a preliminary one, which mainly focused on assessing the biocompatibility and antioxidant activity of a novel formulation of hydrogel in the in vitro models. The stated null hypothesis of the study was there is no statistically significant difference in the biocompatibility and antioxidant activity on using carrageenan-based injectable hydrogel incorporated with different concentrations of *Cissus quadrangularis* extract.

## Methods

### Study and sample characteristics

The present in vitro study was conducted after obtaining ethical approval from the institutional ethical committee (SRB/SDC/ENDO-2105/21/034). Depending on the con-centration of the *Cissus quadrangularis* aqueous extract, the current study had four study groups as mentioned below:

- Group I: Carrageenan hydrogel (without any addition of *Cissus quadrangularis*, considered as control group (n=15);
- Group II: Carrageenan hydrogel with 10% w/v of *Cissus quadrangularis* aqueous extracts (n=15)
- Group III: Carrageenan hydrogel with 20% w/v of *Cissus quadrangularis* aqueous extracts (n=15),
- Group IV: Carrageenan hydrogel with 30% w/v of *Cissus quadrangularis* aqueous extracts (n=15).

### Sample size calculation

Considering the invitro nature of the study, the post-hoc power of the study was calculated using G Power software. The effect size of 1.70 was calculated based on the mean recorded in the current study for the variables tested in the study. The alpha error was kept at 0.05 for a study with four groups. Each group had a sample size of 15, making the total sample size of sixty. With the above input values the power of the study was 1.

### Preparation of hydrogel

**Fig. 1:**
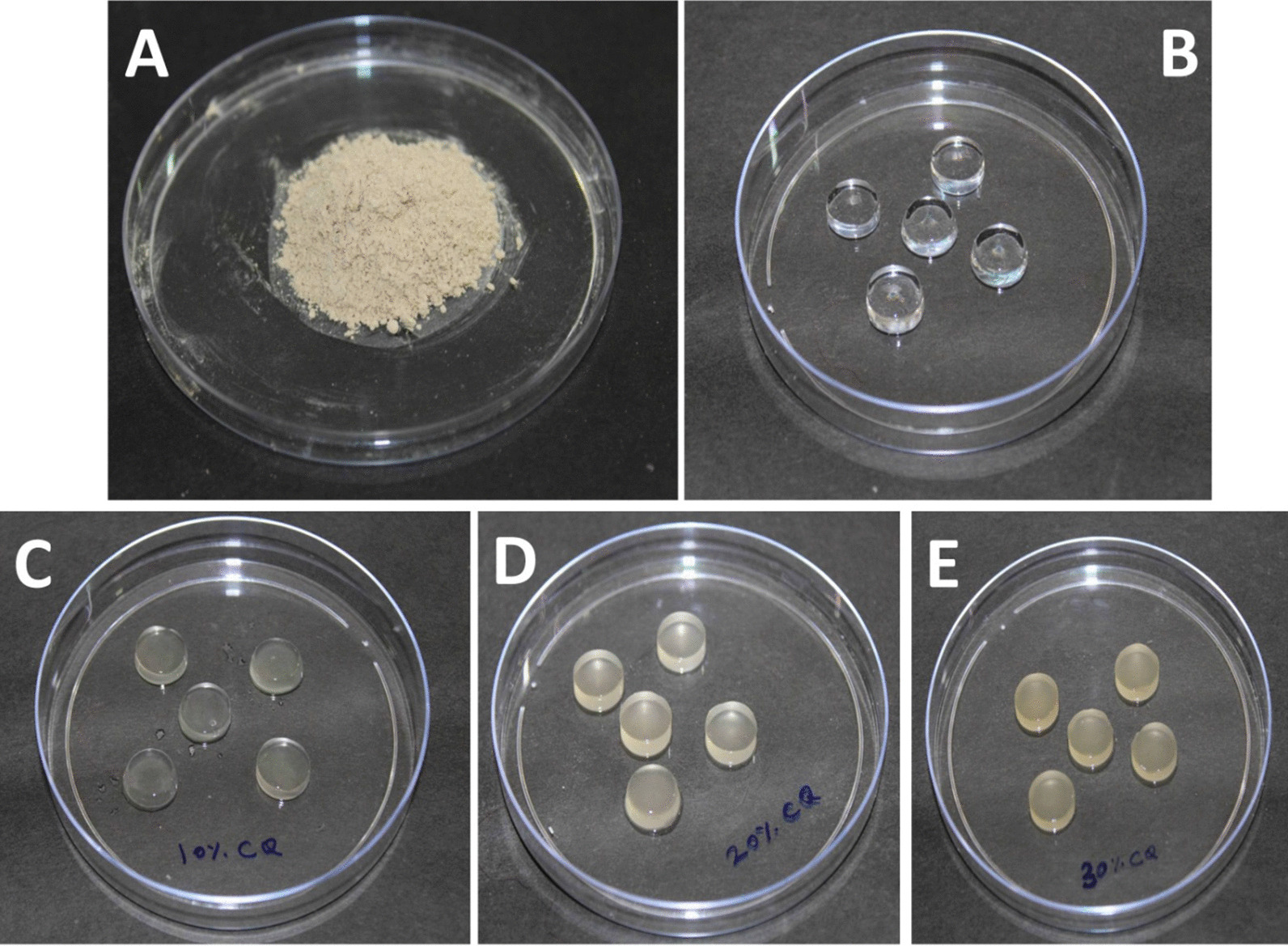
**A** Commercially available *Cissus quadrangularis* powder; **B** Carrageenan hydrogel (control); **C** 10% w/v *Cissus quadrangularis* hydrogel; **D** 20% w/v *Cissus quadrangularis* hydrogel; **E** 30% w/v *Cissus quadrangularis* hydrogel

Commercially available Carrageenan powder (Tokyo Chemical Indrustries (TCI), CASReg no: 11114-20-8, MeronTM, India), *Cissus quadrangularis* powdered extract (Annai Aravindh Herbals®, ISO 9001:2015 Certified SKU-AAH_PH_S_PROI, Chennai, India) and distilled water were used to prepare the hydrogel. A 100 mL of distilled water was heated at 60 °C for 30 min and 0.5 g of commercially available carrageenan powder was then dissolved in it by continuous stirring to make the carrageenan hydrogel. It served as the control agent in the study. A 10 g, 20 g, and 30 g of commercially available *Cissus quadrangularis* powdered extract were then dissolved in 100 mL of distilled water to produce 10% w/v, 20% w/v and 30% w/v aqueous extract of *Cissus quadrangularis* respectively. A 100 mL of this aqueous extract was then added to 0.5 g commercially available carrageenan powder and continuously stirred at 60 °C to form 10%, 20%, 30% w/v *Cissus quadrangularis* hydrogel [23]. The prepared hydrogel was then poured into standard moulds of dimension 6 × 2 × 2 mm^3^ and stored at 4 °C Before testing, the prepared hydrogel was sterilized using autoclaving for 15 min at 121 °C at 15 psi (Fig. 1A–E).

### Testing for antioxidant activity: DPPH radical scavenging assay [24]

The prepared samples of the hydrogels were then subjected to antioxidant assay using a 1,1-diphenyl-2-picrylhydrazyl (DPPH) model system. A 50 µL of control hydrogel and different concentrations (10%, 20%, 30% w/v) of *Cissus quadrangularis* hydrogel were taken in test tubes. Later, 1 mL of a 0.1 mM methanolic solution of DPPH and 450 µL of 50 mM of TrisHCl buffer (pH 7.4) were added and incubated for 30 min. Later, the reduction in the quantity of DPPH free radicals was assessed depending on the absorbance at 517 nm. The butylatedhydroxytoluene (BHT) was employed as a control. The percentage of inhibition was determined from the following equation

$${\text{Percentage}}\,{\text{of}}\,{\text{inhibition}} = \frac{{{\text{Absorbance}}\,{\text{of}}\,{\text{control}} - {\text{Absorbance}}\,{\text{of}}\,{\text{test}}\,{\text{sample}} \times 100}}{{{\text{Absorbance}}\,{\text{of}}\,{\text{control}}}}$$

### Testing for biocompatibility: brine shrimp lethality assay [25]

A 2 g of iodine-free salt was weighed and dissolved in 200 mL of distilled water. Fifteen, 6 well enzyme-linked immunoassay (ELISA) plates were taken and 10–12 mL of saline water was filled. Ten live nauplii were slowly added to each well. The wells were labelled as control, 10%, 20% and 30% according to the concentrations *Cissus quadrangularis* aqueous extract. A 50 µL of each concentration of hydrogel were added to each well as per their respective concentration and plates were incubated for 24 h. Later, the ELISA plates were observed and noted for the number of live nauplii present and calculated by using the following formula

$${\text{Percentage}}\,{\text{of}}\,{\text{live}}\,{\text{nauplii}} = \frac{{{\text{Number}}\,{\text{of}}\,{\text{live}}\,{\text{nauplii}} \times 100}}{{{\text{Number}}\,{\text{of}}\,{\text{live}}\,{\text{nauplii}} + {\text{number}}\,{\text{of}}\,{\text{dead}}\,{\text{nauplii}}}}$$

### Microstructure and surface morphology analysis using scanning electron microscopy

The microstructure and surface morphology of the hydrogel samples containing different concentrations of *Cissus quadrangularis* aqueous extract was investigated using SEM analysis. The hydrogel samples were stored at − 50 °C for 48 h and dried in a lyophilizer (Virtis Benchtop 4k freeze dryer). The cross-sectional surfaces of the sample in powder form were coated with a thin layer of platinum sputter and then SEM analysis was performed using field emission scanning electron microscopy (FESEM IT800).

### Statistical analysis

The gathered data was entered into the MS excel sheet. The normality of the data was assessed using the Shapiro–Wilk test. Data was presented in mean ± standard deviation and considering the parametric nature of data, One-way ANOVA with Post Hoc Tukey test was performed to derive inferential statistics. The data was analysed using IBM (IBM Corporation Business Analytics) SPSS software 23.0 version.

## Results

### Comparative analysis of antioxidant activity among study groups

**Fig. 2:**
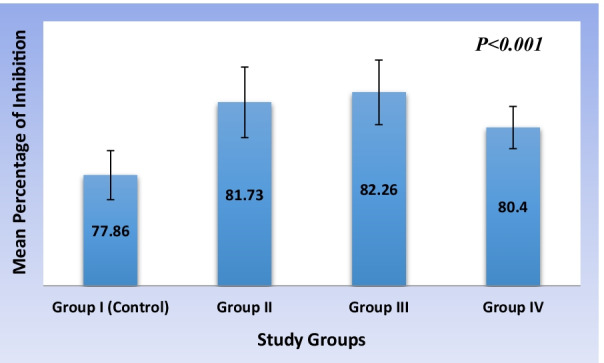
Comparative analysis of antioxidant activity among the study groups

A statistically significant (*P* < 0.001) difference in the antioxidant activity was observed among the study groups with control (group I) and group III reported with the least and highest antioxidant activity (Fig. 2). On post-hoc evaluation, the group I exhibited significantly (*P* < 0.001) least antioxidant activity compared with other study groups. There was a non-significant (*P*˃0.05) gradual increase in the antioxidant activity between group II and III. On the other hand, a significant (*P* < 0.01) drop in the activity was recorded with increased concentration of aqueous extract of *Cissus quadrangularis* hydrogel from 20% w/v (group III) to 30% w/v (group IV) (Table 1).

**Table 1:** Post-hoc evaluation of antioxidant activity among study groups

Pair-wise comparison groups	Mean difference	P value	95% confidence interval	
			Lower bound	Upper bound
*Group I*				
Group II	− 3.867	.000^¶^	− 5.35	− 2.39
Group III	− 4.400	.000^¶^	− 5.88	− 2.92
Group IV	− 2.533	.000^¶^	− 4.01	− 1.05
*Group II*				
Group I	3.867	.000^¶^	2.39	5.35
Group III	− .533	.776	− 2.01	.95
Group IV	1.333	.092	− .15	2.81
*Group III*				
Group I	4.400	.000^¶^	2.92	5.88
Group II	.533	.776	− .95	2.01
Group IV	1.867	.008^€^	.39	3.35
*Group IV*				
Group I	2.533	.000^¶^	1.05	4.01
Group II	− 1.333	.092	− 2.81	.15
Group III	− 1.867	.008^€^	− 3.35	− .39

Group I: Carrageenan hydrogel (without any addition of *cissus quadran-gularis*; Group II: Carrageenan hydrogel with 10% w/v of *Cissus quadrangularis* aqueous extracts; Group III: Carrageenan hydrogel with 20% w/v of *Cissus quadrangularis* aqueous extracts; Group IV: Carrageenan hydrogel with 30% w/v of *Cissus quadrangularis* aqueous extracts; Intergroup comparison was carried out with one-way ANOVA, which showed a statistically significant difference in antioxidant activity

^¶^p < 0.001; ^€^P < 0.01

### Comparative analysis of biocompatibility among study groups

**Fig. 3:**
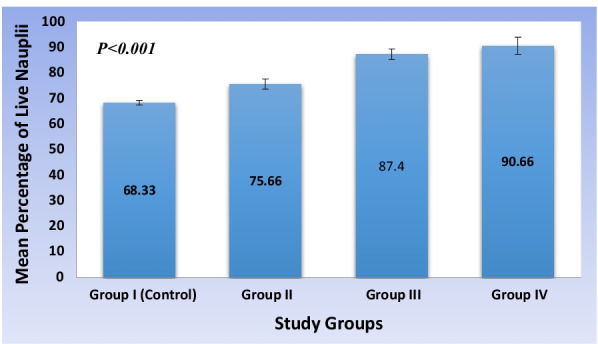
Comparative analysis of biocompatibility among the study groups

A statistically significant (*P* < 0.001) increase in the biocompatibility was observed among the study groups with group I and group IV reported with the least and highest level of biocompatibility (Fig. 3). On post-hoc evaluation, a significant (*P* < 0.001) increase in the biocompatibility was observed with each increment increase in concentration of aqueous extract of *Cissus quadrangularis* hydrogel (Table 2).

**Table 2:** Post-hoc evaluation of biocompatibility among study groups

Pair-wise comparison groups	Mean difference	P value	95% confidence interval	
			Lower bound	Upper bound
*Group I*				
Group II	− 7.333	.000^¶^	− 9.52	− 5.15
Group III	− 19.067	.000^¶^	− 21.25	− 16.88
Group IV	− 22.333	.000^¶^	− 24.52	− 20.15
*Group II*				
Group I	7.333	.000^¶^	5.15	9.52
Group III	− 11.733	.000^¶^	− 13.92	− 9.55
Group IV	− 15.000	.000^¶^	− 17.18	− 12.82
*Group III*				
Group I	19.067	.000^¶^	16.88	21.25
Group II	11.733	.000^¶^	9.55	13.92
Group IV	− 3.267	.001^€^	− 5.45	− 1.08
*Group IV*				
Group I	22.333	.000^¶^	20.15	24.52
Group III	15.000	.000^¶^	12.82	17.18
Group IV	3.267	.001^€^	1.08	5.45

Group I: Carrageenan hydrogel (without any addition of *Cissus quadrangularis;* Group II: Carrageenan hydrogel with 10% w/v of *Cissus quadrangularis* aqueous extracts; Group III: Carrageenan hydrogel with 20% w/v of *Cissus quadrangularis* aqueous extracts; Group IV: Carrageenan hydrogel with 30% w/v of *Cissus quadrangularis* aqueous extracts; Intergroup comparison was carried out with one-way ANOVA, which showed a statistically significant difference in biocompatibility

^¶^p < 0.001; ^€^P < 0.01

### Microstructure and surface morphology analysis using scanning electron microscopy

**Fig. 4:**
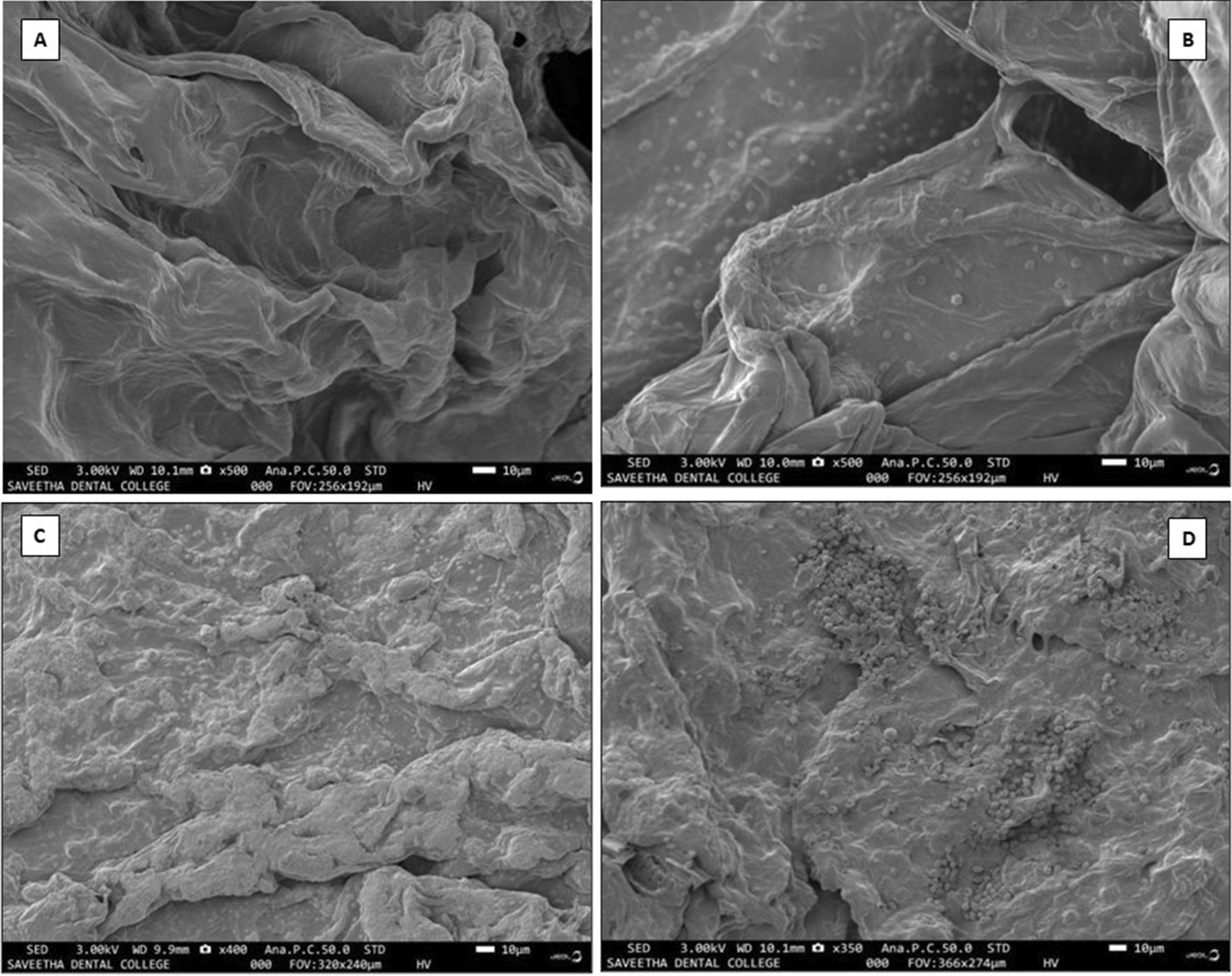
**A** Porous microstructure of carrageenan hydrogel. **B** 10% w/v aqueous extract of *Cissus quadrangularis* hydrogel showing evenly dispersed particles of *Cissus quadrangularis* **C** 20% w/v aqueous extract of *Cissus quadrangularis* hydrogel showing evenly dispersed dense arrangement of *Cissus quadrangularis* particles. **D** 30% w/v aqueous extract of *Cissus quadrangularis* hydrogel showing clumping of *Cissus quadrangularis* particles

**Fig. 5:**
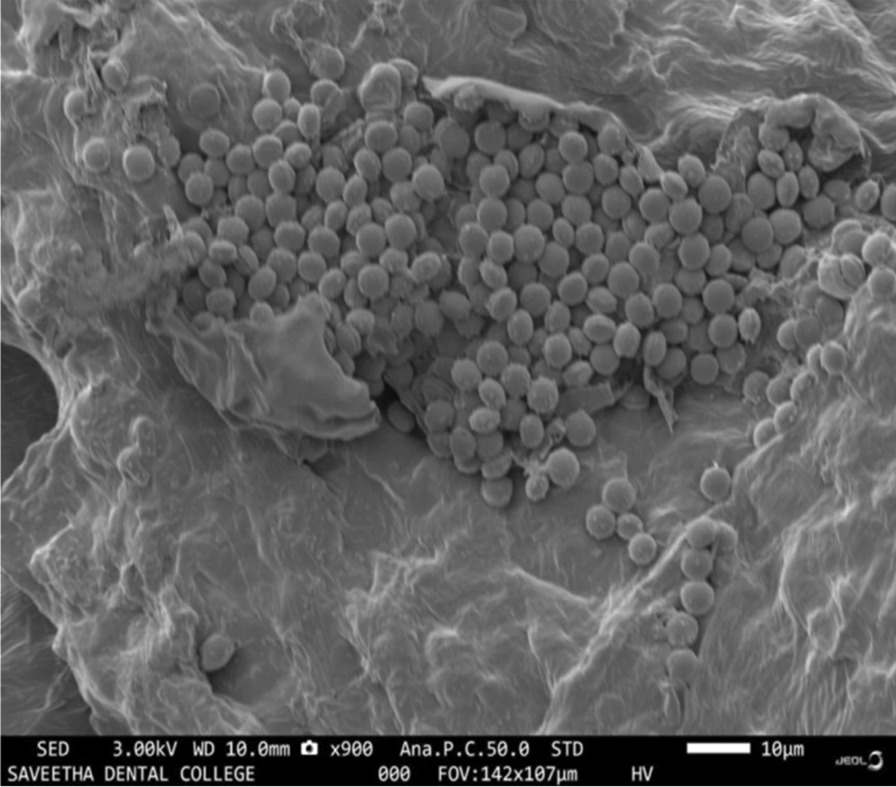
Higher magnification view of 30% w/v *Cissus quadrangularis* hydrogel (Group IV) with evident clumping of *Cissus quadrangularis* particles

The SEM analysis of the control group revealed a porous structure of the hydrogel. It is essential for the regeneration as it provides a matrix for the stem cells of the apical papilla to embed and differentiate further into odontoblasts to lay down the dentin. In group II (10% w/v aqueous extract of *Cissus quadrangularis* hydrogel), an even distribution of *Cissus quadrangularis* was seen throughout the hydrogel, whereas in group III, in addition to even distribution *Cissus quadrangularis*, the particles were shown to be close and densely arranged. The group IV, showed an uneven distribution of *Cissus quadrangularis* particles with the particles being clumped together (Figs. 4, 5).

## Discussion

In recent years, research is ongoing to determine the use of hydrogel as a scaffold for regenerative procedures. Although many studies have focused on the use of scaffolds in tissue engineering, reports are scarce on the use of injectable carrageenan hydrogel scaffolds infused with *Cissus quadrangularis*. Hence, as a preliminary study, we focused on assessing the biocompatibility and antioxidant property of the *Cissus quadrangularis* incorporated carrageenan-based hydrogel scaffold. A patent under the World intellectual property organization, with international publication no—WO 2008/081233 A2, with the inventor/applicant AVESTHA, GENGRAINE TECHNOLOGIES PVT LTD states that the percentage yield of whole plant extracts with active ingredients was the highest in water which was about 17.31%. The rest of the solvents used for extraction were hexane, 80% ethanol and acetone whose yields were much lesser than that of water. In the current study, this was the rationale for using aqueous extract of *Cissus quadrangularis* for the preparation of hydrogel and for further tests conducted [26].

In the present study, the rationale for selecting the carrageenan hydrogel lies in the fact that it enhances favourable results in the tissue regeneration process. This hydrogel has superior mechanical properties that depend on its molecular weight, source, concentration, type and degree of cross-linkage [27]. The molecular weight of the hydrogel influences its degradation property. It has been found that as the molecular weight increases the degradation rate decreases [28]. Studies showed that the degradation rate was higher when a lower-weight scaffold such as chitosan-based hydrogels was used [29]. Furthermore, the three-dimensional structure of carrageenan has shown osteoblastic proliferation and adhesion [30]. A combination of carrageenan hydrogels with different delivery systems has shown successful outcomes [31]. The prerequisites for an injectable hydrogel include flowability under low pressure, rapid setting at the target site and preserving the appropriate integrity and strength [32]. Due to its emulsifying and thixotropic property, it could be used as an injectable scaffold [33].

In the present study, we have used a carrageenan-based hydrogel incorporated with various concentrations of *Cissus quadrangularis* extracts which is a bioactive compound. To the best of our knowledge, the latter has not been explored for its therapeutic potential; however, its potential for osteogenesis has been extensively studied. The osteogenesis potential of *Cissus quadrangularis* extract has been explored in various dental clinical situations such as periodontal bone regeneration [34], mandibular alveolar ridge distractions [35] and in maxillofacial [36] and mandibular fractures [37]. In particular, the *Cissus quadrangularis* extracts have been shown to contain calcium, along with other compounds [38] and thus probably have shown to regulate osteoblastic activity [39] by enhancing osteoblastogenesis [39], mineralization [40] and eventually induce bone formation for faster bone healing [41]. Literature also showed that *Cissus quadrangularis* extract stimulated the mineralised nodules in dental pulp cells [42].

Recent literature found the stage-specific and tissue expression of BSP [43], and OPN [43] in reparative dentinogenesis. On the other hand, studies are showing the efficacy of *Cissus quadrangularis* extract on OPN [44], bone morphogenetic protein (BMP) [45] and BSP [46] activation, which in turn gives us an idea of the usage of this current material as a scaffold for tissue engineering, as the material has immense potentiality for osteogenesis and mineralisation.

In the present study, *Cissus quadrangularis* and carrageenan hydrogels are combined to assess antioxidant activity and biocompatibility. To date, there are no reports on the usage of combinations of these injectable hydrogels for assessing these properties. The reason for choosing only 10–30 weight/volume incorporations of *Cissus quadrangularis* extracts was based on the results from the previous study [39], which showed maximum bone healing at 10gm/100 ml. Additionally, multiple observations in the prior experiments of SEM of the present lab showed maximum antioxidant activity with evenly distributed extract throughout the hydrogel on 20% w/v *Cissus quadrangularis*. Observations above concentration 30% w/v showed clumping, hence, in the present study, it was restricted to 30 w/v concentration extract. Considering the above observations, the above-mentioned incorporations of cissus extracts in the hydrogel were considered in the present study.

Reactive oxygen species are well recognised for their dual role play. Although they might play constructive in cell physiology, they may also cause the destruction of cell membranes and DNA [47, 48], which could be deleterious to the regenerative process. They cause significant damage to the cell structures during oxidative stress. They are cytotoxic and implicated in the aetiology of various pathological conditions [49]. Hence, antioxidants counteract these reactive oxygen species and thereby reducing the harmful effects induced by them. Especially the antioxidants play an immense role during regeneration by promoting the environment favourable. Hence, before usage of any biomaterial, it’s important also to assess its antioxidant activity. For the assessment of the antioxidant activity, the DPPH test was performed in the present study as it has been popularly used to test the antioxidant properties of the plant extracts. In the DPPH assay, the addition of the extract to a violet-coloured DPPH solution reduces it to a yellow-coloured product, diphenylpicryl hydrazine in a concentration-dependent manner. The convenience offered by the short duration of the assay, allows its wide applications to predict antioxidant activity [50]. Dhanasekaran S et al. (2020) evaluated the antioxidant property of *Cissus quadrangularis* with different concentrations ranging from 25 to 400 μg/mL in ethanolic, and methanolic extracts. In this in vitro model, the free radical scavenging activity was more in methanolic extract compared to ethanolic extract in a dose-dependent manner [24]. In the present study, 10 and 20% w/v *Cissus quadrangularis* showed a significant gradual increase in the antioxidant activity as compared to the control group, however, a significant drop in the activity was observed with a further increase in the concentration of the agent to 30% w/v. On SEM analysis it was observed that 10% and 20% w/v *Cissus quadrangularis* hydrogel showed an even distribution of the incorporated agent throughout the hydrogel but when the concentration is increased to 30% w/v, an uneven distribution with clumping of the *Cissus quadrangularis* particles was noted. This could be a probable reason for the reduced antioxidant activity seen with the increasing concentration.

Any biomaterial used for regeneration, should not elicit any undesirable effects and should perform its desired function with appropriate beneficial cellular or tissue response [51–53]. The success of any biomaterial inevitably depends on its acceptability by the native tissue [54]. Hence, it’s important to evaluate the biocompatibility of the material used for regeneration. To evaluate the same, carrageenan-based hydrogel infused with *Cissus quadrangularis* was subjected to brine shrimp lethality assay. The current study showed a significant increase in biocompatibility with the increasing concentration of the additive agent in the scaffold. *Cissus quadrangularis* extract has been extensively explored for its antioxidant, anti-inflammatory and bone tissue regeneration in various in vitro studies [20]. Previous reports have shown better cytocompatibility of *Cissus quadrangularis* when performed using 3-(4,5-dimethylthiazol-2-yl)-2,5-diphenyl-2H-tetrazolium bromide (MTT) assay for bone tissue engineering [20]. In another study, the least cytotoxicity of carrageenan hydrogels was seen when performed on L929 fibroblast cells [16]. In the present study, *Cissus quadrangularis* and carrageenan hydrogels are combined to assess antioxidant activity and biocompatibility. To date, there are no reports on the usage of combinations of these injectable hydrogels.

### Limitations and future directions

This is a preliminary in vitro study to assess the antioxidant and biocompatible property of carrageenan injectable hydrogel infused with *Cissus quadrangularis*. It’s important to assess the setting time and degradation rate of the material which is intended to be used for regeneration. The current in-vitro study is a preliminary one where we have not focussed on assessing the properties of the prepared hydrogel. The interaction between *Cissus quadrangularis* extract and carrageenan needs to be studied further. Whether the sulphated groups and slightly acidic nature of the carrageenan inhibit the bio-active compounds from *Cissus quadrangularis* is unknown. Further studies need to be done using stem cells from the apical papilla (SCAP) cell line to evaluate the biocompatibility of the hydrogel prepared. Studies need to be performed using organic and polar solvents to see if more active ingredients are released which can be used to synthesise a hydrogel with better antioxidant potential.

## Conclusions

Within the limitation of the study, it can be concluded that 20% w/v *Cissus quadrangularis* infused carrageenan based injectable hydrogel showed enhanced antioxidant activity whereas when the concentration of *Cissus quadrangularis* was increased to 30% w/v, has shown diminished response. However, there was a significant increase in the biocompatibility in a dose-dependent manner.

## Data Availability

The datasets generated and/or analyzed during the present study are not publicly available as ethics approval was granted on the basis that only the researchers involved in the study could access the identified data but are available and accessible from the corresponding author on reasonable request.
